# Conformational Dynamics of Viral Protease Precursors in Maturation, Inhibition, and Drug-Resistance Development

**DOI:** 10.3390/v18090967

**Published:** 2026-09-02

**Authors:** Taťána Majerová, Pavel Novotný

**Affiliations:** 1 Institute of Organic Chemistry and Biochemistry of the Czech Academy of Sciences, 166 10 Prague, Czech Republic; 2 Department of Physical and Macromolecular Chemistry, Faculty of Science, Charles University in Prague, 128 43 Prague, Czech Republic

**Keywords:** viral proteases, protease precursors, autoprocessing, cis cleavage, conformational dynamics, allostery, antiviral drug discovery, drug resistance, polyprotein processing

## Abstract

Viral proteases process viral polyproteins into functional proteins and are therefore essential for viral replication and antiviral drug development. Most research has focused on mature enzymes, whereas precursor and partially processed protease forms remain poorly understood. This review examines viral protease maturation as a dynamic and temporally regulated process. Immature precursors are often membrane-associated and conformationally heterogeneous, and their activation is controlled by mechanisms such as cis cleavage, dimerization, cofactor binding, and interdomain communication. These regulatory steps represent potential vulnerabilities in production of viral progeny. We discuss how conformational dynamics influence protease maturation in coronaviruses, flaviviruses, picornaviruses, and retroviruses, and how defined precursor states may be pharmacologically exploited. Targeting these states could complement inhibition of the mature enzyme, increase the barrier to drug resistance, or dysregulate maturation by inducing premature protease activation, thereby disrupting viral particle production. Protease maturation therefore offers a conceptual framework for antiviral strategies that extend beyond classical active-site inhibition.

## 1. Introduction

Many medically important viruses use a polyprotein strategy in which large polyproteins are cleaved by viral proteases into functional proteins (Table 1). In many cases, the protease itself is embedded within a membrane-associated polyprotein. Autocatalytic release of the protease initiates viral maturation, yet the mechanisms governing autoprocessing remain incompletely understood [1]. Importantly, in some viruses, such as flaviviruses or coronaviruses, host-cell proteases also participate in specific steps of polyprotein processing [2,3,4,5,6].

Viral proteases are established drug targets, and protease inhibitors are used clinically to treat infections caused by HIV, hepatitis C virus, and SARS-CoV-2. However, drug discovery efforts have focused almost exclusively on mature enzymes [7]. Clinically used compounds block viral substrate cleavage at concentrations several orders of magnitude lower than those required to block autoprocessing of the protease precursor. Differences between inhibition of viral-substrate processing and inhibition of autocleavage are linked to the distinct mechanisms of these two events, as discussed below [8,9,10,11]. Targeting proteases in their precursor forms would interfere with the first step of the maturation cascade, potentially amplifying the antiviral effect and increasing the barrier to the development of drug resistance.

Protease precursors likely populate heterogeneous conformational ensembles, only a subset of which is cleavage-competent [1,12,13]. This heterogeneity may contribute to the temporal regulation of activation, preventing both premature and delayed maturation. Shifting the population of conformers can also dysregulate these orchestrated processes. In addition, transient binding pockets may emerge in specific precursor conformations and could potentially be exploited by allosteric small molecules [14]. Such compounds may also act synergistically with active-site inhibitors by prolonging one another’s residence time [15]. Targeting two sites on the same molecule could further reduce the likelihood of resistance development.

**Figure 1:**
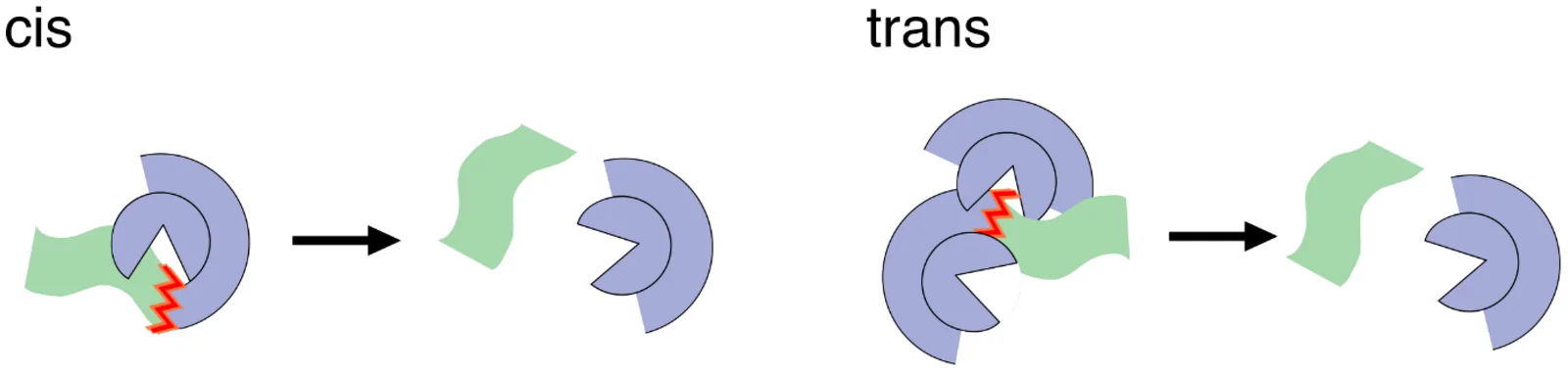
A schematic representation of cis (intramolecular) and trans (intermolecular) autoprocessing. The protease domain is shown in blue, the cleaved polyprotein segment in green, and the cleavage site in red.

Viral proteases are generally regarded to be liberated from their polyprotein precursors by autoprocessing [1,16,17,18,19]. Autoprocessing can occur through cis or trans cleavage (Figure 1). Trans cleavage is conceptually straightforward: one enzyme molecule cleaves a separate substrate molecule; in this context, the substrate is a viral polyprotein that also contains the protease. During trans cleavage, the conformational dynamics of the polyprotein substrate are important determinants of processing efficiency by viral or host proteinases [4,20,21].

By contrast, cis cleavage occurs when the protease cleaves itself from its own polyprotein precursor. This represents a “two-in-one” reaction in which a single molecule acts as both enzyme and substrate. It is therefore a unimolecular reaction and is independent of enzyme concentration. In some viruses, such as HIV, Dengue virus or WNV, the initial autoprocessing steps have been shown to occur exclusively in cis [19,22,23,24], likely because the cleavage site has limited steric accessibility. The exclusive cis cleavage cannot be rescued by trans proteolysis; if this process is impaired, addition of a functional enzyme in trans has no effect [19,25,26]. The main features of exclusive cis and trans cleavage are summarized in Table 2. When cis and trans cleavage can alternate, the outcome depends on their relative contributions.

Many mature viral proteases lack transmembrane regions and function as obligate dimers, with dimerization stabilizing their structure and enhancing catalytic activity (Table 1). By contrast, their precursor forms are often predominantly monomeric and remain anchored to host-cell membranes. Autoprocessing is therefore closely linked to both proper subcellular localization and dimerization-dependent regulation: membrane anchoring confines protease activation to the appropriate compartment, while dimerization governs the formation of catalytically competent precursor states and coordinates subsequent steps in viral maturation [1].

The following sections summarize current knowledge of autoprocessing in selected medically important viruses, the role of protein dynamics in autoprocessing and inhibition, and the potential for modulating autoprocessing with small molecules. We then discuss how these insights may be leveraged to design antivirals with higher barriers to resistance and to develop first-in-class therapeutic strategies.

## 2. Coronaviruses

**Figure 2:**
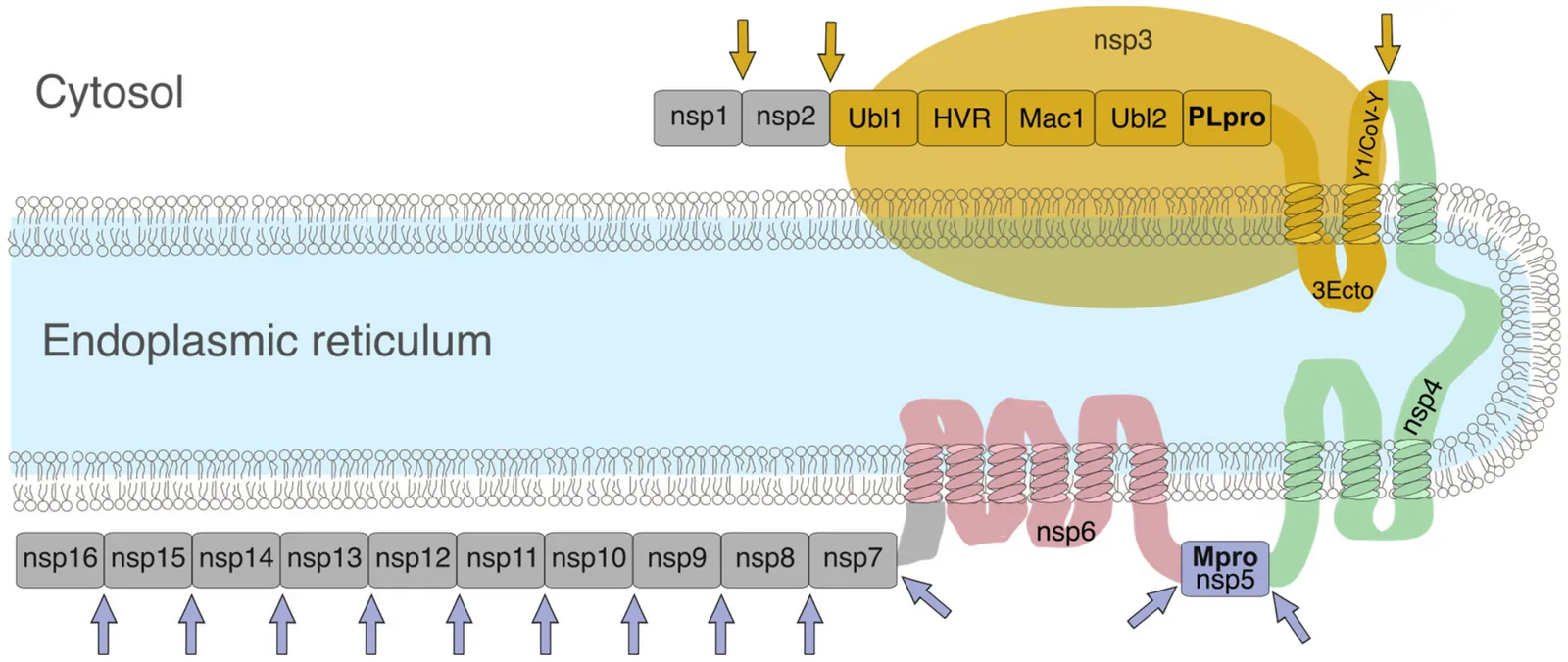
Schematic representation of the uncleaved SARS-CoV-2 polyprotein pp1ab anchored in the ER membrane harboring the protease PLpro (as a part of multidomain nsp3) and one monomer of Mpro together with other nonstructural proteins (nsps). Yellow arrows indicate PLpro cleavage sites, and blue arrows indicate Mpro cleavage sites.

Coronaviruses are enveloped, positive-sense, single-stranded RNA viruses transmitted mainly by respiratory droplets. The best-known examples include severe acute respiratory syndrome coronavirus 2 (SARS-CoV-2), severe acute respiratory syndrome coronavirus (SARS-CoV), and Middle East respiratory syndrome coronavirus (MERS-CoV). Coronaviruses combine discontinuous transcription to generate subgenomic mRNAs encoding structural proteins with polyprotein synthesis to obtain non-structural proteins. Their polyproteins associate with endoplasmic reticulum (ER) membranes and remodel them to form double-membrane vesicles (DMVs). These polyproteins are processed by two polyprotein-embedded cysteine proteases: the papain-like protease (PLpro) within nonstructural protein 3 (nsp3) and the main protease (Mpro) within nonstructural protein 5 (nsp5) (Figure 2). Mpro active-site inhibitors, including nirmatrelvir, simnotrelvir (both co-administered with ritonavir), and ensitrelvir, are approved for clinical use in different parts of the world [7,27].

Interestingly, a rebound phenomenon has been observed in some patients, in whom viral load increased several days after initial clinical improvement and completion of nirmatrelvir treatment [28]. Recent experiments showed that infected cells treated with high concentrations of nirmatrelvir contained multilayered membrane structures and canonical DMVs. After nirmatrelvir washout and continued cultivation of cells, production of infectious virions recovered. This observation may reflect a potential recovery of DMVs after inhibitor removal or the persistence of viral RNA within cells. Effective prevention of post-treatment viral rebound may require elimination of persistent DMVs and residual viral genomic RNA [29]. Hypothetically, if one role of DMVs is to protect viral polyproteins from degradation in the host cell, and if inhibitor-induced changes are reversible after inhibitor withdrawal, Mpro inhibitors with long residence times or compounds capable of perturbing polyprotein structure could reduce the risk of viral rebound. Verifying such hypotheses will require extensive future experimental work.

Both coronavirus proteases are extramembranous enzymes located on the cytosolic side of the endoplasmic reticulum. The initial autocleavage events mediated by SARS-CoV-2 Mpro and PLpro, which release the mature Mpro enzyme and the mature multidomain nsp3 protein, respectively, can occur through both cis and trans cleavage mechanisms [18,30,31]. However, the relative contributions of these pathways in vivo remain unclear.

### 2.1. SARS-CoV-2 PLpro

SARS-CoV-2 nsp3 is a large multidomain protein containing an extensive cytosolic region that includes PLpro, followed by multiple transmembrane helices. Nsp3 is followed by nsp4, another multi-pass transmembrane protein. Cleavage of the nsp2–nsp3 and nsp3–nsp4 junctions by PLpro, as well as cleavage of the nsp4–nsp5 junction by Mpro, generates mature nsp3 and nsp4. Both multidomain proteins remain associated with ER-derived replication membranes through their transmembrane regions. The membrane-associated regions of nsp3 and nsp4 cooperate in the biogenesis of DMVs and assemble into a double-membrane-spanning pore complex proposed to mediate the export of newly synthesized viral RNA from the DMV lumen to the cytosol [32].

In addition, the N-terminal signal-peptide-like region of nsp4 can be partially cleaved by host signal peptidase. This processing generates nsp4 species with a more flexible luminal N-terminal domain, which may facilitate interactions with nsp3 and promote assembly of the nsp3–nsp4 DMV pore [6]. Nsp3–nsp4 cleavage appears to be essential for DMV formation, whereas nsp4–nsp5 cleavage seems to be dispensable [33]. However, the extramembrane C-terminal domain of nsp4, which is approximately 90 amino acids in length, localizes to the lumen of DMVs during DMV formation [32] and regulates the number of DMVs generated [34]. This extramembrane C-terminal domain of nsp4 is conformationally dynamic, and its structural state depends on sequence context, including whether nsp4 remains fused to nsp5. This intrinsic flexibility may create an opportunity to pharmacologically target the nsp4 C-terminus [35]. Ligands interacting with the C-terminal extramembrane helices of nsp4 could also affect Mpro (nsp5) autoprocessing.

PLpro exhibits deubiquitinating activity, which contributes to evasion of host immune responses. Jun12682, a noncovalent SARS-CoV-2 PLpro inhibitor, exhibits antiviral activity in vitro and in a mouse model, including against nirmatrelvir-resistant viral variants. The compound binds the ubiquitin-binding domain of PLpro and does not interact with the human deubiquitinases USP7 and USP14, which are close homologues of PLpro [36]. The earlier noncovalent inhibitor GRL0617 binds the P3 and P4 substrate pockets. In one conformational state, GRL0617 closely contacts PLpro residue Leu 162, whereas in another state, Leu 162 is positioned farther from the inhibitor. This conformational heterogeneity may contribute to the compound’s limited potency [37]. A newer series of PLpro inhibitors binds adjacent to the GRL0617-binding site and engages a previously unrecognized pocket. This pocket may correspond to a low-population dynamic conformation stabilized by ligand binding [38]. Additional biophysical and structural studies are required to test this hypothesis.

Other viruses also encode deubiquitinating proteases embedded within large multidomain proteins. Members of the genus *Orthonairovirus* are unusual among negative-sense RNA viruses because they encode a proteolytic enzyme. In these viruses, the protease is not released by autoprocessing but remains catalytically active within the L protein. A well-characterized example is the ovarian tumor (OTU) protease from Crimean–Congo hemorrhagic fever virus (CCHFV) [39,40,41,42]. This enzyme is highly dynamic, and its conformational plasticity likely enables engagement of multiple host-cell substrates, presumably through a conformational selection mechanism [43]. African swine fever virus (ASFV), a double-stranded DNA virus in the family *Asfarviridae*, provides another example; it encodes a protease belonging to the SUMO-1-specific protease family [44].

Papain-like proteases may be modulated by both active-site and allosteric binders. Their multidomain structures are likely to contain multiple ligand-binding pockets, allowing ligands to affect not only the domain to which they bind but also distal domains through allosteric communication networks. Moreover, papain-like proteases are not limited to viral polyprotein processing; many also cleave host-cell targets, frequently through deubiquitinating or deISGylating activities, thereby suppressing innate immune responses. These properties make papain-like proteases and their precursor forms particularly attractive antiviral drug targets.

### 2.2. SARS-CoV-2 Mpro

Mature SARS-CoV-2 Mpro functions as a dimer, with one active site in each monomer, whereas each viral polyprotein contains a single Mpro monomer (Figure 2). Release of the authentic free N-terminus enables the enzyme to adopt its stable mature tertiary structure [45,46,47]. This highlights the interconnection between autoprocessing and dimerization. The earliest proposed steps of autoprocessing involve N-terminal intramolecular cleavage, resulting in an increased population of dimers, followed by C-terminal processing in trans [48]. However, autoprocessing can occur even in the absence of mature-like dimerization: dimerization-defective Mpro mutants can undergo autoprocessing but lack trans activity [49]. Conversely, N-terminal processing does not appear to be essential for dimerization, as substrate binding can induce dimer formation regardless of the maturation state [50].

Positive modulation of enzyme activity by nsp4- and nsp5-derived peptide substrates, and even by active-site inhibitors under specific conditions, has been observed in vitro [31,51,52,53], whereas other substrates do not induce pronounced allosteric effects [54,55,56]. Because the Mpro homodimer is asymmetric, positive modulation may depend on the ability of a substrate or ligand to engage both protomers simultaneously [54]. Substrate-mediated positive modulation of enzymatic activity, coupled with an increased propensity for dimerization, may also contribute to triggering autoprocessing in vivo.

A proposed model of SARS-CoV-2 Mpro precursor autoprocessing suggests that most precursor molecules adopt inactive E conformations, whereas a minor population adopts an active E* conformation. The E* state predominates in mature Mpro and permits slow initial cis N-terminal autoprocessing [48,57]. Stabilization of inactive E states could therefore represent a potential antiviral strategy [58]. Interestingly, Mpro maturation is much less sensitive to mutations at the N-terminal autoprocessing site than to mutations in trans-cleaved substrates, indicating fundamental differences between cis and trans cleavage mechanisms [31,59]. In Omicron variants, the T492I mutation in the cytosolic C-terminal region of nsp4, adjacent to the nsp4/nsp5 cleavage site, increases Mpro-mediated cleavage efficiency at this junction. This alteration is associated with enhanced viral replication and evasion of innate immune responses but reduced lung pathogenicity [60]. Enhanced suppression of proinflammatory cytokines may contribute to this reduced virulence [61].

Active-site inhibitors stabilize the dimeric form of Mpro and several model precursor forms [62]. At low inhibitor concentrations, this stabilization is accompanied by increased precursor enzyme activity [31,57]. One possible explanation is that the inhibitor binds to one monomer within the dimer, leaving the second monomer free but allosterically activated and competent to cleave substrates. Studies of mature Mpro have shown that both active sites can be occupied simultaneously by small molecules, including in the catalytic mutant C145A. C145A is more stable than wild-type Mpro and enhances activity when it forms a heterodimer with a wild-type monomer [63].

An allosteric communication network connecting the two protomers—comprising Ser10, Ser113, Gly146, Ser147, Gly149, and His163—has been identified. Mutations at these positions reduce catalytic activity and impair dimerization, although ligand binding can restore dimer formation. Mixing C145A with variants that disrupt the allosteric network partially rescues activity, demonstrating that complementary defects can compensate within heterodimers. Overall, dimerization and cooperativity regulate Mpro activity in response to enzyme and substrate concentrations, helping to ensure that activation and cleavage occur at the appropriate time and place [63]. Interprotomer allosteric communication is central to these processes and may represent a target for small-molecule modulation [64,65]. Simultaneous disruption of intraprotomeric and interdimeric interactions has a deleterious synergistic effect on Mpro activity [66].

Dimerization mediates allosteric communication between Mpro subunits and is itself subject to allosteric regulation. Allosteric binding sites have been identified in mature Mpro [67,68,69,70]. The in vivo dimerization propensity of Mpro and its precursor forms is difficult to assess experimentally. For purified mature Mpro, the dimerization constant has been reported to be in the micromolar range and decreases with increasing temperature, suggesting a relatively weak monomer–monomer interaction [71]. Precursor forms may dimerize even less efficiently, although this possibility remains to be demonstrated directly. The modest affinity of the mature dimer suggests that pharmacological disruption of Mpro dimerization may be feasible.

The de novo-designed miniprotein HB3-Core25, a validated dimerization inhibitor, demonstrates the feasibility of this approach. It shifts the monomer–dimer equilibrium toward monomers. Interestingly, HB3-Core25 appears to inhibit Mpro activity only partially, reaching a plateau of approximately 50%, which is not overcome by increasing inhibitor concentration. One possible explanation is that the miniprotein binds only a subset of conformational states [72]. Alternatively, limited solubility or aggregation could reduce the apparent potency. More speculatively, binding to one protomer might create an asymmetric or slowly dissociating dimer in which only one active site remains temporarily competent during enzyme kinetic experiments.

Compounds that shift the Mpro monomer–dimer equilibrium without inhibiting mature Mpro in vitro have nevertheless been reported to exhibit anti-SARS-CoV-2 activity. This antiviral effect has been attributed to interactions with immature Mpro precursor forms [73]. These examples indicate that inhibitor design need not be restricted to the active site of the mature enzyme but could also target dimerization interfaces and allosteric sites in mature enzymes or precursor forms.

In summary, the first step of Mpro autoprocessing is thought to involve intramolecular N-terminal cleavage. Qualitatively, this step is relatively resilient to cleavage-site mutations and current inhibitors, and it does not require mature-like dimerization. However, the strong conservation of autoprocessing sites, together with residues important for dimerization and interprotomer allosteric communication, indicates that Mpro autoprocessing must be tuned during the viral life cycle. This is illustrated by the Omicron-associated T492I mutation in nsp4, which quantitatively alters processing at the nsp4/nsp5 junction by Mpro and directly affects viral replication. Targeting the earliest steps of Mpro maturation could therefore complement conventional active-site inhibition. Potential strategies include compounds that efficiently bind the precursor active site, shift polyprotein conformational ensembles, alter the propensity to form transient dimers, or modulate cleavage-site accessibility. The feasibility of these approaches remains to be explored in future studies.

Despite differences in substrate specificity, a similar architecture—a dimer with one active site per monomer—is observed in the monkeypox virus protease, a cysteine protease expressed in an already active mature form [74,75]. Monkeypox virus I7L protease contains a dynamic “cap” structure that adopts open and closed conformations. Molecular dynamics simulations support the concept that switching between these conformations regulates substrate entry into the active site [76]. Monkeypox virus, together with smallpox and vaccinia viruses, belongs to the *Poxviridae* family, which comprises enveloped double-stranded DNA viruses transmitted by direct contact and droplets [77]. Among other DNA viruses, herpesviruses encode a protease known as assemblin. Assemblin is a homodimeric serine protease with one active site per monomer [78]. It is expressed as part of a protease–scaffold precursor, in which the scaffold region mediates interaction with the major capsid protein and facilitates transport of the complex into the cell nucleus. Association with capsid proteins promotes precursor dimerization and protease activation [79]. Thus, assemblin activity is regulated by protein–protein interactions and associated dynamic conformational changes, which may also represent potential targets for antiviral intervention [1,80,81,82]. Using a conformation-selective antibody, a cryptic allosteric site formed by the latch loop was identified. This site regulates protease dimerization and activation and may be exploited for small-molecule inhibitor discovery [83].

## 3. Flaviviruses

In viruses of the family *Flaviviridae*, the serine protease forms the N-terminal domain of the multifunctional NS3 protein, which also exhibits NTPase and helicase activities. Proteolytic activity requires heterodimerization with an activating cofactor, a short protein expressed as a fusion product either downstream of NS3, as in hepatitis C virus (HCV; genus *Hepacivirus*), or upstream of NS3, as in viruses of the genus *Flavivirus*, including dengue, West Nile, Zika, and tick-borne encephalitis viruses (TBEV) [84]. HCV cleaves substrates with uncharged amino acids at the P1 position, whereas proteases from viruses of the genus *Flavivirus* typically cleave after two consecutive basic residues [85,86,87].

### 3.1. Flaviviruses of the Genus Hepacivirus

HCV is the best-known human-infecting member of the genus *Hepacivirus* and is transmitted primarily through blood-to-blood contact. Other members of this genus infect horses, rodents, and bats [88].

#### 3.1.1. NS3/4A Serine Protease

The heterodimeric HCV NS3/4A protease contains a catalytic serine, adopts a chymotrypsin-like fold, and contains a structural zinc ion [89,90]. Interaction between NS3 and NS4A optimizes the arrangement of the catalytic triad and substrate-binding pocket [91,92]. Although eight major HCV genotypes have been defined, active-site inhibitors of NS3/4A, including glecaprevir and voxilaprevir, are approved HCV treatments. However, individual inhibitors differ in their activity across genotypes [7,93].

**Figure 3:**
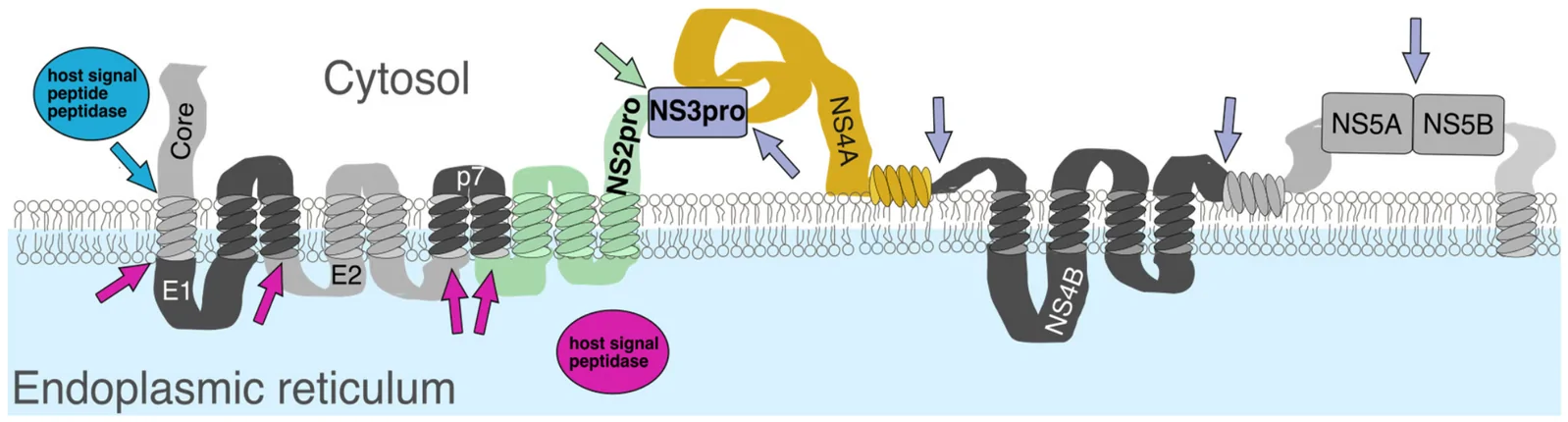
HCV polyprotein containing two protease domains (NS2 and NS3) and the NS4A activating region. Arrows indicate cleavage sites processed by host proteases (blue, green and magenta), the NS2–NS3 protease (green), and the NS3/NS4A protease complex (blue).

In HCV, the NS3–NS4A junction is cleaved exclusively in cis (Figure 3). This cis cleavage is substantially less sensitive to cleavage-site mutations than sites cleaved in trans, suggesting that it is strongly influenced by polyprotein folding [94].

Experimental fusion proteins showed only a twofold difference in activity between NS3 constructs with and without a free N-terminus. Thus, unlike many other proteolytic enzymes, the NS3/4A protease does not require a free N-terminus for activity. By contrast, an uncleavable NS3/4A construct carrying mutations in the NS3–NS4A cleavage site lacked proteolytic activity [95]. On the other hand, a construct containing the entire NS3 protein, including the protease, NTPase, and helicase domains, exhibited higher proteolytic activity than a protease-domain-only construct, indicating allosteric communication between NS3 domains [95].

The protease active site is thought to face the ER membrane. For cis cleavage between NS3 and NS4A, the hydrophilic helicase domain must partially embed into the ER membrane. After cleavage, the helicase domain rotates away from the protease domain and adopts a functionally active extended conformation. Stabilization of the autoinhibited closed conformation of NS3/4A could therefore block polyprotein processing and inhibit formation of the replicase complex. Allosteric inhibitors binding to the protease–helicase interface that stabilize an inactive closed conformation of NS3/4A have been reported [96].

#### 3.1.2. NS2 Cysteine Protease

HCV also encodes NS2, a zinc-dependent cysteine protease (Figure 3). NS2 is an obligate homodimer. Each polyprotein contributes one NS2 monomer to form the dimer, and each active site contains residues from both subunits: one monomer provides the catalytic histidine and glutamate residues, whereas the other contributes the nucleophilic cysteine. NS2 cleaves only the NS2–NS3 junction in cis, thereby triggering subsequent steps required for production of infectious viral particles [97,98,99]. Following cleavage, the newly generated C-termini remain coordinated within both active sites, suggesting product-mediated autoinhibition of the protease [97,100]. NS2 activity is tightly regulated by its interaction with NS3 [98,101]. In addition, NS2 activity is modulated by reversible palmitoylation at Cys113, which stimulates autoprocessing and promotes recruitment of the envelope protein E2 to detergent-resistant membranes [102].

Exclusive cis cleavage represents a marked viral vulnerability because it cannot be restored in trans by another protease. Blocking such a “starter protease” could therefore have particularly detrimental effects. Targeting cis-autoprocessing steps that cannot be rescued in trans represents a promising but still underexplored antiviral strategy [26]. Other viruses, including picornaviruses, encode analogous starter proteases like 2A, whose primary function is an early single cis-autoprocessing event [103].

### 3.2. Flaviviruses of the Genus Flavivirus

Flaviviruses of the genus *Flavivirus* cause febrile, sometimes life-threatening diseases and are transmitted primarily by mosquitoes or ticks.

#### NS2B/NS3 Serine Protease

**Figure 4:**
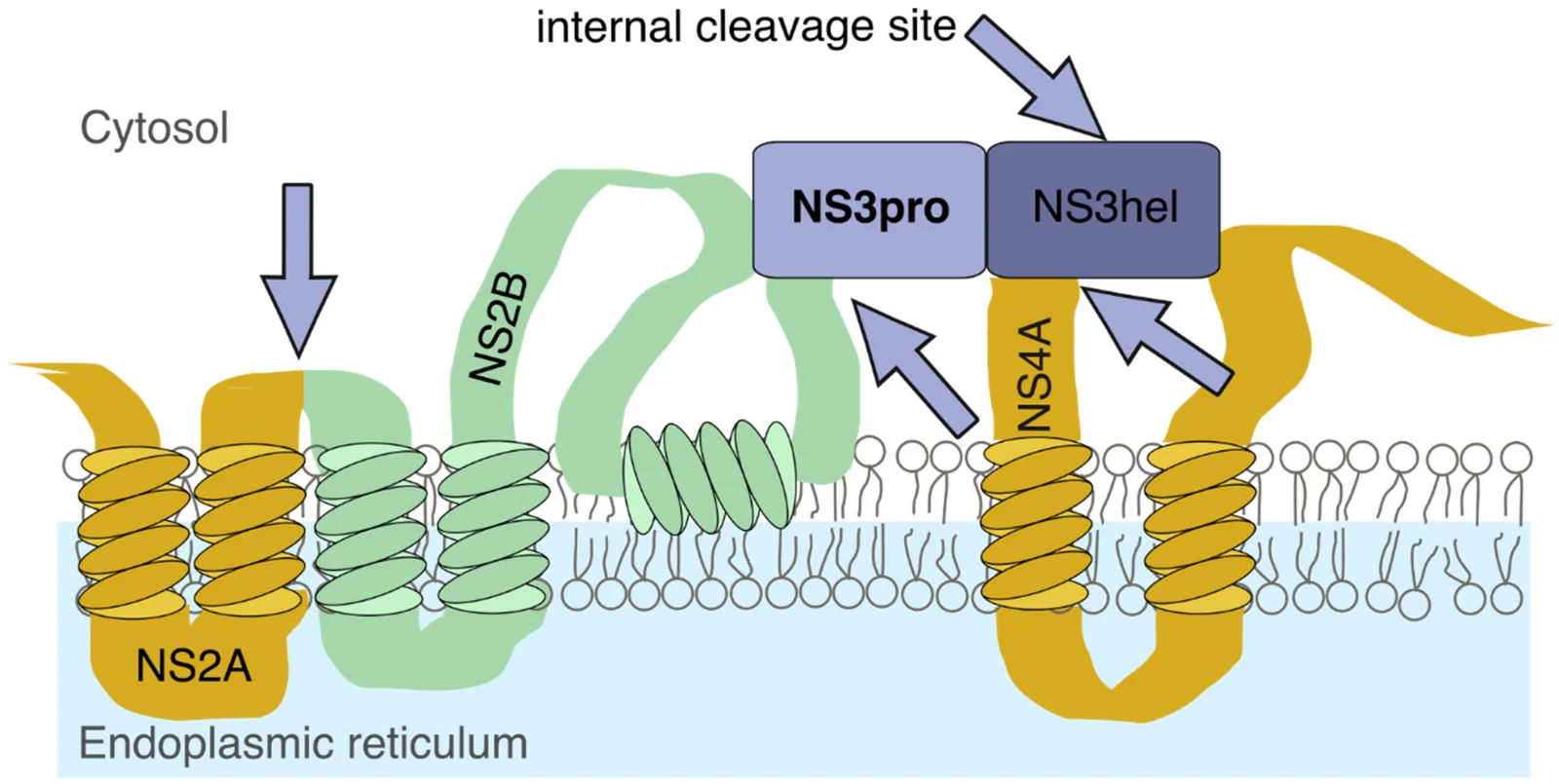
Flaviviral protease precursor anchored in the ER membrane. NS2B/NS3 protease cleavage sites are indicated by blue arrows.

They encode a single heterodimeric protease called NS2B/NS3, a serine protease with a chymotrypsin-like fold (Figure 4). No NS2B/NS3 inhibitor is currently approved for clinical use. NS3 is a multidomain protein that also contains NTPase and helicase domains. JNJ-1802 (mosnodenvir), which blocks the interaction between the NS3 helicase domain and NS4B, prevents formation of the replication complex in all dengue virus serotypes and exhibits potent antiviral activity [104]. Although mosnodenvir entered phase II clinical trials and demonstrated safety and prophylactic efficacy [105], subsequent trials were discontinued due to changes in company priorities [106]. Another potent compound with a similar mechanism, NITD-688, has also been reported [107,108].

Flaviviral proteases are highly dynamic and interconvert between open and closed conformational states [109,110,111,112,113]. NS2B is anchored in the ER membrane by both termini, and its hydrophilic extramembrane region acts as an activating cofactor for NS3 [25,84]. In vitro biochemical studies use artificial protease constructs lacking transmembrane segments. The design of these expression constructs can strongly influence the conformational dynamics of the recombinant protein [114]. The type of construct used in a study should be considered one of the experimental variables, not a technical detail.

In Zika virus, the eZiPro construct, which contains the natural cleavage site between NS2B and NS3, exhibits much lower activity than other constructs [115]. This reduced activity is primarily due to the processed NS2B C-terminus occupying the active site and blocking substrate entry [116], although this inhibition can be overcome by competing inhibitors [117]. A similar phenomenon was observed for the WNV NS2B/NS3 protease [24]. The gZiPro construct, in which NS2B and NS3 are connected by a glycine–serine linker, is more active but conformationally constrained [118]. The most active construct is a bicistronic NS2B–NS3 construct lacking the cleavage-site sequence; this variant is also the most sensitive to aprotinin [116].

No analogous differences in activity were observed among dengue virus serotype 4 constructs [118]. Dengue virus serotype 2 NS2B–NS3 constructs exhibited lower structural heterogeneity than Zika virus constructs [118]. However, in dengue virus, structural heterogeneity persisted even after binding of a covalent inhibitor, indicating the existence of at least two ligand-binding modes that stabilize interconverting conformations [119].

Analogous to the eZiPro construct, viruses of the family *Togaviridae* encode monomeric proteases that are released by autoprocessing. Chikungunya virus is a prominent member of this family. In Venezuelan equine encephalitis virus (VEEV), the cysteine protease nsP2 adopts an autoinhibited conformation after processing, in which its N-terminus occupies the active-site cleft and restricts access by additional substrates. These findings suggest that nsP2 proteolytic activity is regulated by conformational dynamics, enabling condition-dependent control of enzyme activity [120].

Dengue virus serotype 4 NS2B–NS3 constructs containing full-length NS3 revealed that the noncovalently linked heterodimer predominantly adopts a closed conformation, whereas NS2B–NS3 covalently linked by a glycine-rich linker adopts an open conformation. This covalent constraint reduces both protease and NTPase activities [121].

Overall, NS2B–NS3 is a dynamic protein complex that switches between open, closed, and semi-open conformations. Each conformational state may harbor distinct allosteric pockets. Therefore, construct-dependent artifacts must be considered in NS2B–NS3 protease studies, as alterations in conformational dynamics can influence ligand binding [122].

Further complicating this picture, recent work on dengue virus serotype 4 combining cross-linking mass spectrometry (XL–MS), molecular dynamics (MD) simulations, mutagenesis, and protease assays showed that the conserved NS2B residue S48 interacts not only with the protease domain but also with the helicase domain. The authors proposed a compact conformation, not captured in crystal structures, that is enabled by the intrinsic dynamics of the NS2B–NS3 complex [123].

The active site of dengue virus serotype 2 NS2B/NS3 protease is flanked by two conserved loops: R24–G39, containing a hydrophobic tip, and L149–A164, containing a hydrophilic tip. MD simulations revealed bending and rocking motions of these “regulatory fingers” around the active site, with pronounced changes in the presence of DMSO. Enzyme kinetic experiments showed that DMSO primarily affects k_cat_, which also decreases at low temperature. A reduced k_cat_ is consistent with a noncompetitive mechanism that does not directly perturb the active site. Mutations in the loop tips confirmed that coordinated loop motions are required for activity; disruption of these movements reduced enzyme activity. These observations illustrate how conformational plasticity can modulate enzyme function [124].

Dengue virus NS2B/NS3 protease can also bind ligands through conformational selection [125]. Although the protease is active near physiological pH, it undergoes structural rearrangements and may aggregate under acidic conditions. Such conditions can shift the conformational equilibrium and reveal low-population states. Thus, studies of proteases performed under extreme conditions may reveal allosteric binding pockets. Small molecules that preferentially stabilize alternative conformations and shift the conformational equilibrium of the enzyme may perturb protease function and represent a route toward inhibitor design [126]. Because the active site of flaviviral proteases is hydrophilic and resembles those of some human proteases, allosteric inhibition is often considered more promising than direct active-site targeting [127]. MH1, a recent allosteric inhibitor of the Zika virus protease, binds a pocket adjacent to the active site. This interaction stabilizes the open, apo-like conformation by sterically blocking transition to the closed state and disrupting critical NS2B–NS3 interactions. The NS3 Ala125Cys mutation in the proposed binding pocket markedly reduces inhibitory potency [128]. Another pocket is exploited by IRBM-Z-2, a compound active in the nanomolar range [129].

Autocatalytic cleavage between NS2B and NS3, as well as internal cleavage within the NS3 helicase domain, occurs exclusively intramolecularly (in cis). Proper formation of the protease active site does not require cleavage between NS2B and NS3 and therefore does not require a free NS3 N-terminus. Cis cleavages enable structural rearrangements that shift the conformational ensemble [130]. One alternative cis cleavage site has been reported in studies of dengue virus serotype 4 NS2B–NS3 protease in vitro [117].

The atypical compound ARDP0006 is only a weak inhibitor of the mature protease in vitro but exhibits unexpectedly strong antiviral activity against dengue virus in cell culture. ARDP0006 preferentially inhibits cis autoprocessing at the internal NS3 cleavage site. Because of steric constraints, this cleavage apparently cannot be rescued in trans, leading to accumulation of uncleaved precursors that impair downstream steps in the viral life cycle. Consistently, co-transfection of wild-type virus with a cis-cleavage-defective G459L mutant, which blocks the same internal cleavage site in the absence of inhibitor, dominantly suppresses viral RNA synthesis. This experimental setup models a cellular context in which correctly processed proteins, analogous to drug-resistant variants, coexist with cleavage-defective precursors, analogous to drug-susceptible forms. These findings suggest that inhibitors targeting obligate cis-processing events can both block viral replication and suppress the emergence of resistant variants through trans-dominant effects [19].

## 4. Picornaviruses

**Figure 5:**
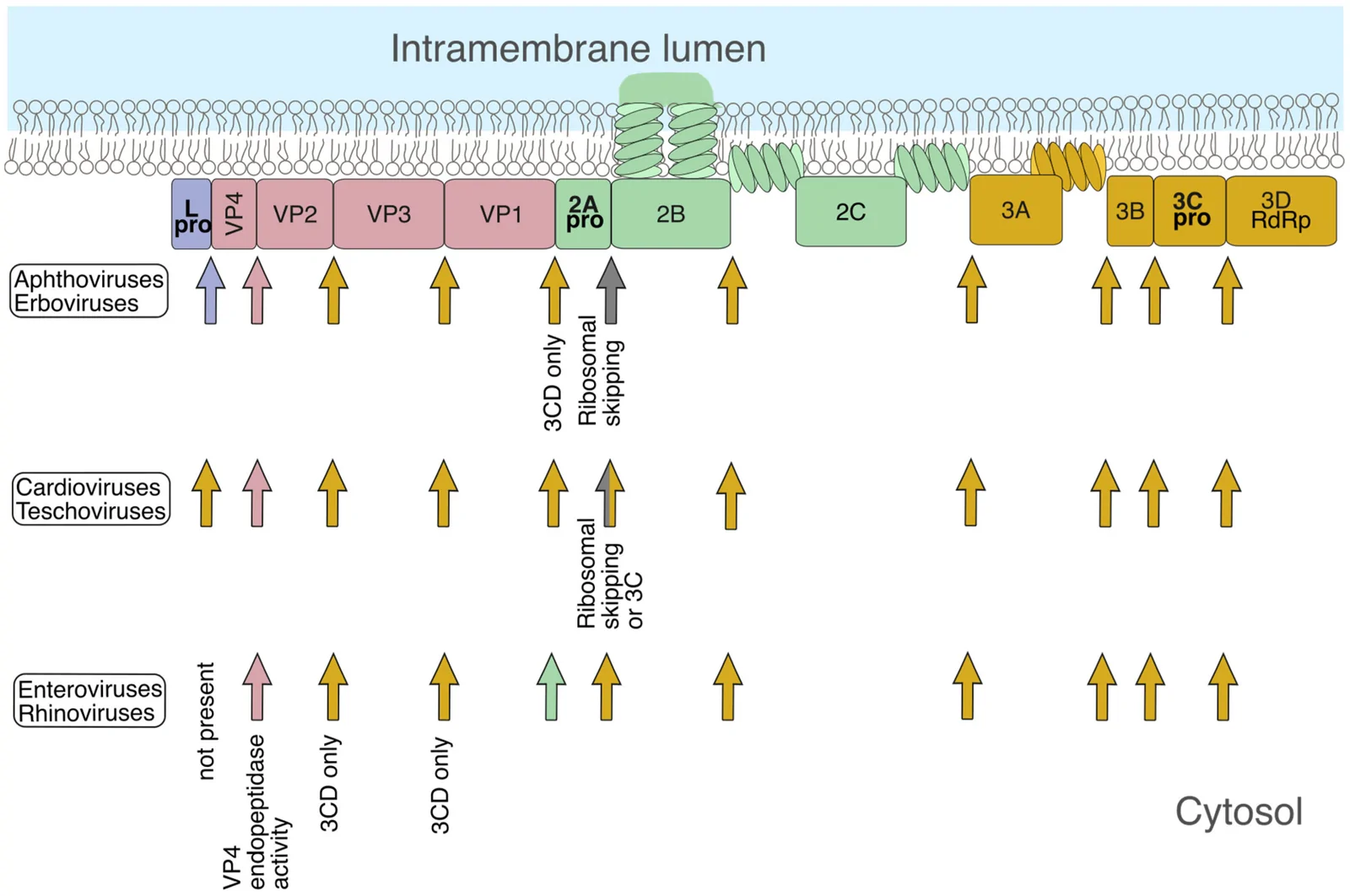
Schematic representation of picornaviral Lpro, 2A and 3C proteases in the context of the polyprotein precursor. The L protease cleavage site is indicated by a blue arrow, 3C-mediated cleavage sites by yellow arrows, 2A-mediated cleavage sites by green arrows, VP4 endopeptidase cleavage sites by pink arrows, and sites of ribosomal skipping by grey arrows.

Picornaviruses, such as rhinoviruses and poliovirus, provide further examples of the polyprotein-based strategy, in which viral proteins are initially expressed as a single precursor and subsequently released by proteolytic processing. The polyprotein encodes two proteases: 2A, located in the P2 region (mentioned above), and 3C, located in the P3 region. The P3 region also encodes 3A, 3B/VPg, and 3D, the RNA-dependent RNA polymerase. In addition to fully processed mature proteins, several cleavage intermediates have distinct functions during infection [131,132]. Aphthoviruses and erboviruses encode L proteins with proteolytic activity at the N-terminus of their polyprotein [133] (Figure 5).

### 3C Cysteine Protease

The 3D domain of the 3CD precursor lacks polymerase activity, whereas the 3C protease domain remains active and may even exhibit greater catalytic efficiency in the 3CD form than as the mature 3C protein [134]. Structural studies show that the overall architectures of 3CD and the isolated 3C and 3D proteins are highly similar, with no major rearrangements of the catalytic sites [135]. The functional differences are therefore attributed to altered conformational dynamics [136,137]. Picornaviral 3C protease interacts not only with proteins but also with phosphoinositide-containing membranes through its positively charged N-terminal helix; it also possesses an RNA-binding region spanning residues 80–95 [138]. Additionally, binding of 3C protease to RNA can induce liquid–liquid phase separation (LLPS) [139]. The multiple processing states and interaction partners of picornaviral proteases provide diverse opportunities for small-molecule modulation beyond classical active-site inhibition.

3ABC is not merely a passive intermediate in polyprotein processing. Although 3ABC cleaves small peptide substrates at rates similar to mature 3C protease (3C^pro^), RNA strongly enhances its ability to cleave larger polyprotein substrates. Thus, RNA elements localized within replication centers may selectively stimulate precursor protease activity at specific cleavage sites [140].

Another precursor, 3CD, binds the 5′ untranslated region (UTR) of the viral genome at the cloverleaf structure through the 3C protease domain. The structure of the 3C–cloverleaf ribonucleoprotein complex shows that 3C recognizes the sugar–phosphate backbone of cloverleaf RNA, which serves as a scaffold for 3C dimer formation. 3CD binds the cloverleaf structure with higher affinity than 3C alone. These findings identify 3C as an unusual RNA-binding protein and provide a structural basis for designing compounds that block ribonucleoprotein assembly [141].

The picornaviral polyprotein is anchored at several sites to membranes of replication organelles derived from the ER–Golgi system [142]. Cleavage between 3A and 3B by 3C protease occurs exclusively in cis and requires host factors, including the sterol/PI4P transporter, which modulates membrane properties, and 3D, the viral RNA polymerase, which interacts with 3AB. 3D can also function when supplied in trans [143]. This highly regulated complex system represents another potential target for small-molecule intervention.

In foot-and-mouth disease virus (FMDV), an important veterinary picornavirus, a mutation that enhances trans cleavage at the 3B–3C junction abolishes replication. The mutation changes the ratio of partially cleaved precursor molecules and produces non-native intermediates. This finding highlights the need for precise temporal control of picornaviral processing and illustrates that both inefficient and excessive polyprotein cleavage can impair viral progeny production [144].

Taken together, picornaviral 3C protease is active not only as a mature enzyme but also in semi-processed precursor forms. These forms have distinct functions, interaction partners, and presumably conformational ensembles. Targeting this precisely regulated machinery with small molecules could therefore represent an attractive antiviral strategy.

A similar architecture, specifically a monomeric protease with a catalytic cysteine in its active site, is found in adenoviral protease. Adenoviruses are DNA viruses that can cause respiratory infections, including common cold-like illness. Their protease is expressed as a mature enzyme but requires activation by viral DNA and by an activating peptide generated through proteolytic cleavage of the viral precursor protein pre-pVI [145,146]. Binding of the activating peptide allosterically triggers a branched sequence of conformational changes, resulting in structural rearrangements that optimize catalytic activity [147]. Treatment of virus-infected cells with a synthetic activating peptide reduced virion production, indicating that inappropriate upregulation of viral protease activity is deleterious for viral replication [1,148].

## 5. Retroviruses

Retroviruses are enveloped, positive-sense, single-stranded RNA viruses whose replication cycle involves reverse transcription of viral RNA into DNA, followed by integration into the host genome [7]. During the retroviral replication cycle, Gag and Gag–Pol polyproteins are translated from the same mRNA. Their ratio is determined by an mRNA stem–loop structure that controls the frequency of a one-nucleotide ribosomal frameshift. Only the Gag–Pol polyprotein contains the viral protease [149]. Gag assembles into an immature lattice at the plasma membrane, where viral components, including Gag–Pol, further modulate protease accessibility, virion release, and the timing of maturation [150]. Assembled virions are enriched in Gag–Pol relative to the cytoplasmic Gag:Gag–Pol ratio. This enrichment reflects nonrandom incorporation during assembly and is facilitated, at least in part, by co-translational interactions between Gag and Gag–Pol produced from the same mRNA. Lower levels of incorporated Gag–Pol are well tolerated and can retain infectivity, whereas elevated levels impair infectivity by promoting premature protease activation. Excess Gag–Pol at assembly sites is limited by inefficient incorporation of Gag and Gag–Pol produced from separate mRNA molecules [151].

### HIV-1 Protease

HIV-1 protease is an obligate homodimer. Each monomer contributes one half of the active site, which is formed at the dimer interface. Although the mature protease has a nanomolar dimerization constant under laboratory conditions, its precursor, Gag–Pol, contains a single protease monomer and is predominantly monomeric [1,16,152,153]. Autoprocessing must therefore be initiated by transient dimerization of the precursor [153,154]. Release of the authentic HIV protease N-terminus triggers conformational rearrangement and stabilizes the mature fold [155].

The first three cleavage events within the Gag–Pol polyprotein occur most probably exclusively in cis. Recent work suggests that this cis mechanism functions primarily as an initiation step because only a minority of Gag–Pol molecules can dimerize. Once dimerization occurs, cis cleavage sites become inaccessible to trans cleavage; in monomeric precursors, however, these sites remain accessible in trans [154].

HIV-1 protease autoprocessing is considerably more tolerant of mutations than mature trans activity. Only the catalytic triad (Asp-Thr-Gly), the dimerization domain formed by the N- and C-termini, and the flap tips (residues 49–52) appear to be indispensable for autoprocessing. Flap motions contribute to the ordered cleavage of the viral polyprotein by modulating the probability of productive cleavage events [156]. Targeting flap dynamics therefore represents a viable strategy for inhibitor design [157,158]. Some mutations in the flap region increase the catalytic activity of mature protease [152,159], although the flaps generally exhibit low mutational tolerance. Deep mutational scanning identified 220 mutations that permit autoprocessing but abolish trans activity. These results support the hypothesis that the precursor exists as a dynamic conformational ensemble, with some regions likely exhibiting intrinsic disorder [160]. Autoprocessing can also be influenced by mutations in other proteins fused downstream of the protease within Gag–Pol, including the C-terminal domains of HIV-1 integrase and RNase H [161,162].

Clinically used HIV-1 protease inhibitors, as well as other experimental compounds, inhibit precursor forms much less efficiently than mature enzymes. An HTS campaign directed against the HIV-1 protease precursor identified several compounds that blocked autoprocessing at concentrations of approximately 40 μM, achieving effects comparable to those of 10 nM darunavir. In virological assays, these compounds exhibited EC_50_ values of approximately 10 μM, whereas darunavir displayed an EC_50_ below 1 nM. The newly identified compounds retained activity against darunavir-resistant mutants. Although their potency remains modest, they represent promising starting points for further optimization [163].

The structure of the HIV-1 Pol polyprotein carrying the protease active-site mutation (D25A) was determined by cryo-electron microscopy (cryo-EM). The polyprotein contains a well-defined core formed by the reverse transcriptase (RT) region, which adopts a dimeric, enzymatically competent configuration even before proteolytic processing. This RT core drives dimerization of the entire Pol assembly. The protease domain, which precedes the RT N-terminus, remains largely inactive in this polyprotein form but retains the ability to dimerize. Its density is poorly resolved, consistent with substantial conformational flexibility. In contrast, the integrase domain at the C-terminus of the polyprotein is largely disordered, suggesting that proteolytic maturation is required for its structural stabilization and function. RT dimerization appears to promote protease proximity and activation, thereby regulating the onset of maturation [164].

Consistent with this model, high-resolution X-ray structures of protease mini-precursors containing short N-terminal extensions show that residues involved in dimer formation adopt a partially ordered state. In these structures, the canonical inter-subunit hydrogen bonds characteristic of the mature protease are not fully established, and alternative interactions are observed, reflecting an immature dimer interface [165]. Similarly, the X-ray structure of a protease–reverse transcriptase fusion protein from simian foamy virus, another retrovirus, indicates that substantial conformational rearrangements are required to achieve the mature enzyme structures [166].

**Figure 6:**
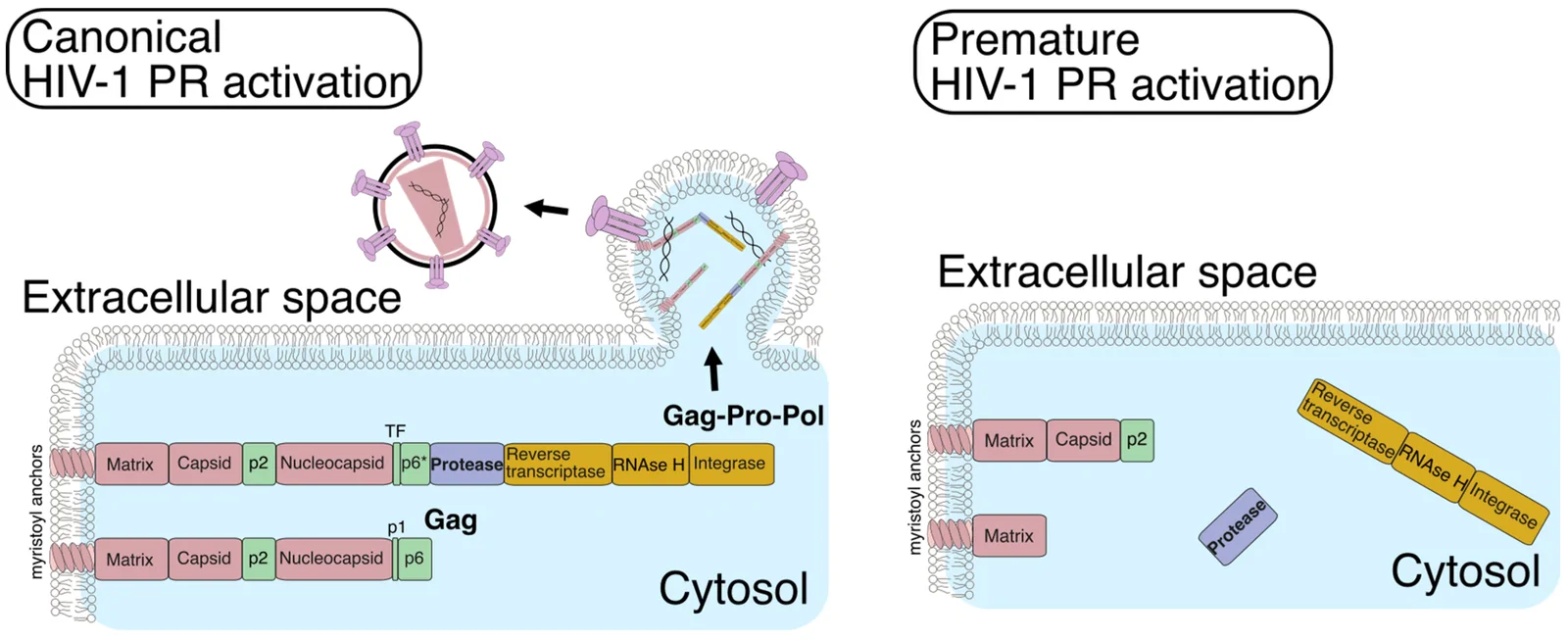
Simplified scheme of canonical and premature HIV-1 protease activation. During canonical activation, Gag and Gag–Pol polyproteins are N-terminally anchored to the host-cell membrane and incorporated into nascent virions. If HIV-1 protease is activated prematurely within the host cell, the protease and other polyprotein fragments are released into the cytoplasm, impairing viral budding. Infected cells may then be eliminated through HIV-1 protease-mediated cytotoxicity. The p6* indicates the frameshift site.

HIV-1 Gag and Gag–Pol polyproteins carry an N-terminal myristoyl group that targets them to the host-cell membrane before viral assembly and budding [167]. The RT domain of Pol plays an important role in Gag–Pol dimerization [168]. An alternative antiviral strategy is to induce premature HIV-1 protease activation by promoting Gag–Pol dimerization. Certain non-nucleoside reverse transcription inhibitors (NNRTIs) bind the RT domain within Gag–Pol, increase its propensity to dimerize, and thereby trigger premature protease autoprocessing (Figure 6) [1,7,169,170,171,172,173,174,175]. Under normal conditions, protease activation is coupled to tightly coordinated assembly events [176,177,178]. Premature protease activation causes earlier and partially mislocalized protease activity within infected cells. Intracellular protease activity is sensed by the CARD8 inflammasome, leading to pyroptotic death of infected cells [7,173,179,180,181,182]. Because HIV integrates into the host genome, this approach could help eliminate infected cells harboring proviral DNA without the need to manipulate the host-cell genome.

Human T-cell leukemia viruses (HTLVs), like HIV, are retroviruses that employ a similar polyprotein-processing strategy [183]. Compared with HIV-1 protease, HTLV-1 protease exhibits lower mutational tolerance during autoprocessing [184]. Because HTLV manifests in long-term chronic infection, strategies that induce premature protease activation, analogous to those explored for HIV, may warrant investigation. However, major immunological challenges, including the risk of excessive inflammation, would need to be carefully addressed [182].

## 6. Role of Conformational Dynamics in Development of Drug Resistance

Drug-resistant protease variants bind inhibitors less efficiently while retaining sufficient capacity to cleave the viral polyprotein. Resistance mutations can reduce not only inhibitor binding but also enzyme activity, necessitating additional compensatory mutations that restore catalytic function while maintaining resistance [7,185,186]. Drug-resistance mutations can occur directly within the substrate-binding pocket or at distal sites that indirectly affect ligand binding by altering the conformational ensemble [187].

The binding free energy of an enzyme–inhibitor interaction includes contributions from protein–ligand contacts, conformational changes in both partners, and solvent effects. High affinity corresponds to a more favorable (more negative) free-energy change and may arise from favorable enthalpic contributions, favorable entropic contributions, or both. Two compounds may have similar overall affinity yet possess markedly different enthalpic and entropic signatures. In principle, affinity can therefore be improved through either enthalpic or entropic optimization [188].

One strategy for designing inhibitors with a higher barrier to resistance is based on the “substrate envelope” concept, which relies on high-resolution structures of natural substrates bound to the enzyme. The substrate envelope defines the volume occupied by natural substrates. Inhibitors should maximize interactions within this envelope while minimizing contacts outside it, except for interactions with catalytic or highly conserved residues or solvent-exposed ligand groups [186,187,188,189,190]. Designing inhibitors with a high barrier to resistance requires maximizing hydrogen bonds with the protein backbone and conserved residues while minimizing contacts with non-conserved residues that can mutate readily [187]. Hydrogen bonds can improve binding enthalpy, but they often restrict conformational freedom of the protein or ligand, creating an unfavorable entropic penalty. This enthalpy–entropy trade-off can limit or even offset the expected gains in affinity [188]. Although ligand binding usually restricts motion at the binding site, distant buried residues can become more flexible and partially compensate for this entropic penalty [191,192,193].

Whether an analogous “precursor substrate envelope” or a maturation-state-specific interaction profile can be defined remains unclear. However, extending inhibitor design toward compounds capable of binding multiple enzyme forms could increase the barrier to the development of drug resistance. Some viral resistance strategies involve changes in the dynamics of the targeted protein. Rigorous characterization of inhibitor binding modes and the dynamic behavior of target enzymes is therefore essential for elucidating the structural determinants of efficient inhibition and for rationally prioritizing compounds for subsequent optimization and development [194,195,196,197].

For example, the naturally occurring SARS-CoV-2 Mpro variant Asp48Tyr/ΔPro168 has increased dynamics in the substrate-binding pockets, resulting in a more open conformation, lower thermal stability, and unexpectedly enhanced dimerization. This variant displays lower affinity for nirmatrelvir and ensitrelvir, whereas GC373 binds with affinity comparable to wild-type Mpro. For GC373, an unfavorable entropy change is compensated by a favorable enthalpy contribution, preserving overall affinity. By contrast, nirmatrelvir and especially ensitrelvir exhibit less favorable binding enthalpy toward the variant, and favorable entropy is insufficient to compensate for this loss. As a result, these inhibitors bind weakly to the mutated variant than to wild-type Mpro. Increased dynamics likely impair formation of critical hydrogen bonds between the mutant protease and inhibitors, leading to unfavorable enthalpic effects [198].

Another example is the different inhibition profiles of SARS-CoV-2 and swine acute diarrhea syndrome coronavirus (SADS-CoV) Mpro, which likely reflect differences in conformational dynamics. Increased flexibility in the 45–51 region of SADS-CoV Mpro is associated with weaker binding of inhibitor h27 (higher K_i_), but also with a higher k_inact_ (the maximal first-order rate constant for covalent inactivation). This suggests that, once a productive enzyme–inhibitor complex forms, covalent bond formation may proceed more rapidly; the higher K_i_ is therefore partly offset by the favorable k_inact_ [199].

Similarly, lower inhibitor affinity for precursor forms may also be dynamically driven. Even mature Mpro samples have multiple conformational states. Ligands that preferentially stabilize nonproductive conformations and shift the equilibrium away from active states could therefore represent useful therapeutic alternatives [200]. Although their X-ray structures are similar, mature SARS-CoV Mpro is more dynamic than SARS-CoV-2 Mpro [201], and SARS-CoV Mpro exhibits lower catalytic activity [46]. At the whole-virus level, SARS-CoV-2 is less pathogenic and more infectious due to complex, multifactorial reasons [61,202].

A mutation-sensitive surface cluster of SARS-CoV-2 Mpro comprising Arg131, Asp197, Asn203, Asp289, and Glu290 lies at the dimer interface and may represent a drug-binding site with a potentially higher barrier to resistance [203]. High-throughput cryo-crystallography combined with covariance analysis of correlated Cα fluctuations across large crystal-structure datasets identified the conserved residue Asn214 as a covariance hotspot. Asn214 participates in a hydrogen-bonding network linking the N-terminus (S1, G1) of one monomer to Phe140 of the opposing monomer, thereby coupling the dimer interface to the active-site loop. Targeting or perturbing this allosteric network may support inhibitor design with an increased barrier to resistance [204]. Three potential allosteric pockets have been independently identified, including the dimer-interface site containing Asn214 [205].

Similarly, many HIV protease drug-resistance mutations do not produce obvious structural changes but instead alter protein dynamics, particularly flap motions [206,207,208]. The L76V mutation, for example, confers resistance to clinically used inhibitors, including darunavir. It does not directly alter the active site; rather, it changes hydrophobic interactions within the protease core and slows precursor maturation by reducing the propensity to form transient dimers. The compensatory M46I mutation restores efficient autoprocessing while preserving impaired inhibitor binding [209]. Designing inhibitors that block the initial transient formation of HIV precursor dimers could therefore represent an alternative antiviral strategy [165,210].

These examples show that drug resistance can emerge not only from the direct loss of inhibitor contacts but also from shifts in the distribution of conformational states. This may be especially important for protease precursor forms, whose expanded conformational ensembles are expected to reduce sensitivity to inhibitors optimized for mature enzymes. The ability of an inhibitor to bind precursor forms that presumably sample multiple conformations could therefore provide an estimate of its conformational tolerance. Such conformational tolerance may, in turn, increase the barrier to the development of drug resistance.

## 7. Outlook

Targeting viral proteases remains a proven antiviral strategy. Currently approved inhibitors primarily bind the active sites of mature proteases and remain vulnerable to ongoing resistance development, driven by high viral mutation rates and incomplete inhibition of resistant variants. A more detailed understanding of early steps in the viral life cycle could open new avenues for antiviral discovery. Here, we have focused on protease autoprocessing in the context of viral maturation and antiviral design.

Disrupting early autoprocessing could collapse the maturation cascade at an irreversible initiation step. Autoprocessing typically begins in cis, when a precursor or zymogenic protease cleaves one or both of its own termini to enable formation of the mature fold and downstream functions. In some systems, autoprocessing is coupled to protease dimerization, which itself may represent a druggable vulnerability.

When autoprocessing begins exclusively in cis (e.g., in HIV protease, flaviviral proteases, picornaviral proteases), it cannot be rescued by alternative pathways, making this step a pronounced vulnerability in the viral life cycle. Protease precursors are generally more conformationally dynamic than mature enzymes, which complicates inhibitor design. However, this same flexibility may also expose additional binding-competent states and, in some cases, reduce entropic penalties if ligand or protein flexibility is retained in the bound state. Allosteric ligands can shift precursor conformational ensembles, and inhibitors capable of binding multiple target conformations may impose higher barriers to resistance. Targeting multiple binding sites on the same enzyme may also produce synergistic inhibition. In dengue virus, accumulation of uncleaved precursors in infected cells can block drug-resistant variants in trans, thereby suppressing the production of resistant progeny.

Conversely, premature activation can be as deleterious as inhibition because it triggers proteolysis in inappropriate cellular contexts. Defining and exploiting these effects may enable noncanonical antiviral strategies.

Testing compounds against precursor forms of viral proteases could help identify inhibitors with broader conformational tolerance and potentially higher barriers to resistance. In addition to inhibitors, positive modulators may also have therapeutic potential; therefore, compounds with such activity emerging from screening campaigns should not be overlooked.

## Figures and Tables

**Table 1:** Examples of medically important viral proteases and their basic properties.

Protease Type	Dimer	Number of Active Sites per Dimer	Viral Family	Example
Aspartate	Yes	1	*Retroviridae*	HIV-1 protease HTLV-1 protease
Cysteine	Yes	2	*Coronaviridae* *Flaviviridae*, genus *Hepacivirus* *Poxviridae*	SARS-CoV-2 Mpro MERS-CoV Mpro HCV NS2–NS3 protease Mpox virus core protease
Cysteine, papain-like	No	–	*Coronaviridae* *Matonaviridae* *Nairoviridae*	SARS-CoV-2 PLpro MERS-CoV PLpro Rubella virus protease Crimean–Congo hemorrhagic virus OTU protease
Cysteine	No	–	*Picornaviridae* *Togaviridae* *Adenoviridae*	Poliovirus 3Cpro Poliovirus 2Apro Chikungunya virus nsP2 protease Adenovirus protease
Serine	Yes	1	*Flaviviridae*	Dengue virus NS2B–NS3 protease Zika virus NS2B–NS3 protease West Nile virus NS2B–NS3 protease HCV NS3/4A protease
Serine	Yes	2	*Herpesviridae*	HSV-1 protease

**Table 2:** Key differences between exclusive cis and trans cleavage.

Feature	Cis (Intramolecular)	Trans (Intermolecular)
Enzyme and substrate are separate molecules	No	Yes
Depends on enzyme concentration	No	Yes
Can be rescued by addition of enzyme	No	Yes
Sensitivity to mutations	Lower	Higher

## Data Availability

No new data were created or analyzed in this review. Data sharing is not applicable to this article.

## Abbreviations

3CLpro—3C-like protease; ASFV—African swine fever virus; CARD8—caspase recruitment domain-containing protein 8; CCHFV—Crimean–Congo hemorrhagic fever virus; COVID-19—coronavirus disease 2019; DENV—dengue virus; DMV—double-membrane vesicle; DMSO—dimethyl sulfoxide; Ex—envelope glycoprotein x; EC_50_—half-maximal effective concentration; EM—electron microscopy; ER—endoplasmic reticulum; FMDV—foot-and-mouth disease virus; Gag—retroviral group-specific antigen polyprotein; Gag–Pol—retroviral Gag-Pol polyprotein, HCV—hepatitis C virus; HIV—human immunodeficiency virus; HTLV—human T-cell leukemia virus; HTS—high-throughput screening; HVR—hypervariable region; ISG15—interferon-stimulated gene 15, LLPS—liquid–liquid phase separation; Mac—macrodomain; Lpro—leader protease; MD—molecular dynamics; MERS-CoV—Middle East respiratory syndrome coronavirus; Mpro—main protease; NMR—nuclear magnetic resonance; NNRTI—non-nucleoside reverse transcriptase inhibitor; nsp—nonstructural protein; NTPase—nucleoside triphosphatase; p7—small hydrophobic protein (viroporin); PLpro—papain-like protease; Pol—retroviral polymerase polyprotein; PR–RT—protease–reverse transcriptase fusion protein; RdRp—RNA-dependent RNA polymerase; RNase H—ribonuclease H; RT—reverse transcriptase; RTC—replication–transcription complex; SADS-CoV—swine acute diarrhea syndrome coronavirus; SARS-CoV—severe acute respiratory syndrome coronavirus; SARS-CoV-2—severe acute respiratory syndrome coronavirus 2; SAXS—small-angle X-ray scattering; SUMO—small ubiquitin-like modifier; TBEV—tick-borne encephalitis virus; TM—transmembrane domain; Ubl—ubiquitin-like; UTR—untranslated region; USP—ubiquitin-specific protease; VEEV—Venezuelan equine encephalitis virus; VP—viral protein; VPg—viral protein genome-linked; WNV—West Nile virus; XL–MS—cross-linking mass spectrometry; Y1—Y1 domain (a conserved domain of unknown function); CoV-Y—CoV-Y domain.
